# Contents under pressure: vascular proliferation and gliosis in the brains of Cavalier King Charles Spaniels with syringomyelia

**DOI:** 10.3389/fvets.2026.1878599

**Published:** 2026-09-09

**Authors:** Christian Atkins, Clare Rusbridge, Silvia Guil-Luna, Pablo Díaz-Santana, Fernando Constantino-Casas, Francisco J. Salguero

**Affiliations:** 1 School of Veterinary Medicine, University of Surrey, Guildford, United Kingdom; 2 Department of Anatomy and Comparative Pathology and Toxicology, School of Veterinary Medicine, University of Córdoba, Córdoba, Spain; 3 Department of Veterinary Medicine, University of Cambridge, Cambridge, United Kingdom

**Keywords:** arteriole, astrocyte, astrogliosis, brain, Cavalier King Charles Spaniel, Chiari-like malformation

## Abstract

Vascular and astroglial changes in the central nervous system of Cavalier King Charles Spaniels (CKCS) affected by Chiari-like malformation (CM) and secondary syringomyelia (SM) and ventriculomegaly (VM) are poorly characterised. CM is a developmental malformation of brachycephalic breeds and is a significant welfare concern, especially for CKCS in which this disorder is considered ubiquitous with varying degrees of severity. A frequent sequela of CM is SM and VM, causing a range of neurological clinical signs. Nineteen deceased CKCS diagnosed with CM were donated for the purposes of research, from which two groups were selected for examination: those with SM and VM (*n* = 13) and those without SM and VM (*n* = 6). Periventricular tissue samples were collected, processed routinely for histology, and stained with H&E, Martius Scarlet Blue (MSB), and glial fibrillary acidic protein (GFAP). Dogs with SM and VM demonstrated increased angiogenesis (23.62 ± 6.79 vs. 11.67 ± 4.93 mean blood vessels per 100x magnification field), and increased vessel wall thickness (71.61% ± 5.14 vs. 58.73% ± 6.47 of total vessel diameter) and greater astrocyte activation, indicated by increased GFAP immunoreactivity, what could be associated with chronic pressure trauma and hypoxic insult resulting from altered vascular compliance. These findings provide insight into the pathological mechanisms associated with CM and its sequelae and may inform future investigations into diagnostic biomarkers and therapeutic targets.

## 1 Introduction

Chiari-like malformation (CM) is a developmental malformation characterised by disproportional neuroparenchymal and skeletal development affecting the skull, craniocervical junction and the adjacent regions of the central nervous system. It is predominantly a disease of miniaturised brachycephalic breeds, in which affected dogs possess a relatively large brain within a reduced cranial cavity (1). Morphological features include reduced caudal cranial fossa capacity with cerebellar herniation and craniocervical malformations (2, 3). CM is considered analogous to Chiari malformation in humans (4). Syringomyelia (SM), defined as a fluid-filled cavity within the spinal cord, and ventriculomegaly (VM), characterised by enlargement of the cerebral ventricles, commonly occur in association with CM and result from disturbed cerebrospinal fluid (CSF) dynamics (5). Although CM may be associated with pain, considerable overlap exists in magnetic resonance imaging (MRI) findings between clinically affected and unaffected dogs, limiting diagnostic discernment based on imaging techniques alone (1). This is compounded by the subjective nature of pain and the variability on its recognition by caregivers and veterinary professionals (6–8). CM is almost ubiquitous in Cavalier King Charles Spaniels (CKCS) (9–11), and common in other toy and brachycephalic dogs (12, 13) and cats (1).

Studies examining the pathogenesis of CM and SM in large mammals are scarce, largely due to difficulties in post-mortem material retrieval (14). In humans, most published accounts are sporadic case reports and case series, with observational datasets of Chiari malformation and associated syringomyelia involving highly heterogeneous human populations (15–19). By contrast, CM and SM are highly prevalent in brachycephalic toy breeds and occur within a homogeneous population, allowing robust comparative analyses and improve investigation of disease pathogenesis. The Cavalier Tissue Collection Scheme, a volunteer-led initiative, supports post-mortem tissue collection from CKCS to support research into major inherited disorders (14, 20, 21). Previous neuropathological investigations have focused primarily on the spinal cord and dura mater, while cerebral pathology remains poorly characterised. (14, 22, 23).

Chronic hydrocephalus is associated with altered cerebral perfusion and subsequent vascular remodelling, astroglial activation and angiogenesis, changes thought to represent adaptive responses to chronic tissue compression and hypoxic injury (24–29). Similar histopathological changes, including angiogenesis, astrocytosis and fibrous proliferation of within arteriolar walls have been documented in the spinal cord parenchyma adjacent to syrinx cavities in CKCS (14). However, corresponding changes in the cerebral periventricular parenchyma of CKCS with SM have not yet been reported.

Adaptive vascular and glial responses described in hydrocephalus mirror histopathological changes reported in spinal cord parenchyma adjacent to syrinx cavities in CKCS (14, 25, 26, 29). These similarities support a shared neurovascular pathophysiology underlying VM and the SM (24, 30). However, it remains unclear whether comparable changes occur within the cerebral parenchyma. This study aims to characterise the vascular and astroglial alterations in the periventricular brain tissue of CKCS affected by CM with long-standing SM and VM.

## 2 Materials and methods

### 2.1 Case selection

Deceased Cavalier King Charles Spaniels diagnosed with CM, part of a retrospective cohort study, were donated for the purposes of research via The Cavalier Collection Scheme (31). Of the initial pool of 26 dogs with CM, 2 groups of cadavers were identified, those with SM and VM (*n* = 13) and those without SM and VM (*n* = 6). Dogs exhibiting only one of the two sequelae, and therefore not meeting the criteria for either study group, were excluded from the analysis (*n* = 7). For example, VM may occur in dogs with CM-associated pain despite the absence of SM.

### 2.2 Tissue processing

**Figure 1:**
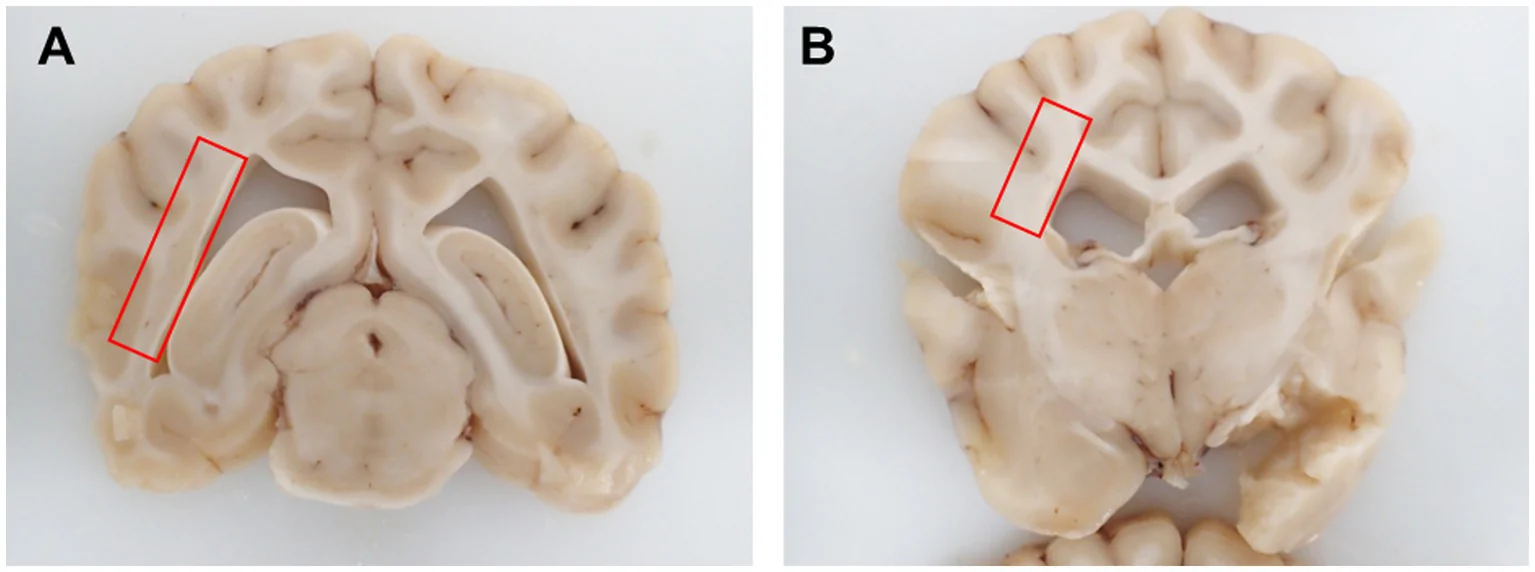
Regions of interest from the cerebral isocortex selected adjacent to the lateral ventricles at rostral **(A)** and caudal **(B)** levels for histological processing.

Central nervous system samples, including the encephalon and the spinal cord were dissected and subsequently fixed in 10% neutral buffered formalin for at least 1 week for optimal fixation and process for histology. Cerebral tissue adjacent to the lateral ventricles was sampled (Figure 1), placed in cassettes and submitted for routine histopathological processing and embedding in paraffin. 4-μm thick sections were obtained and stained routinely with either Haematoxylin and Eosin (H&E) or Martius Scarlett Blue (MSB) for histopathological evaluation.

Sequential sections were also stained by Immunohistochemistry (IHC) for Glial Fibrillary Acidic Protein (GFAP) detection as described previously (32, 33). Briefly, immunostaining was performed on the Leica BOND-RXm (Leica Microsystems) using BOND Epitope Retrieval solution 1 (ER1, pH 6.0) for 20 min at 95 °C as antigen retrieval. After primary antibody (polyclonal anti-GFAP, ThermoFisher Scientific, Mass, United States, diluted at 1:1000) incubation, immunostaining was carried out with the Bond polymer refine detection kit (Leica Microsystems). Finally, slides were counterstained with Harris haematoxylin, routinely dehydrated and mounted using the Dako mounting medium (Agilent, Santa Clara, United States). Appropriate positive control tissues from the archive (dog brain FFPE tissue blocks from the Pathology laboratory at the University of Surrey School of Veterinary Medicine) and negative controls (OMITs) were used in each IHC run.

### 2.3 Histopathological examination

Stained slides were scanned using a Hamamatsu 360 scanner (Hamamatsu, Tokyo, Japan) and e-slides were evaluated using viewing software ndp.view2.

Cerebral tissue adjacent to the lateral ventricles was examined histologically. These slides underwent morphometric and digital analysis using the Nikon-NIS-Ar software.

(Nikon, Tokyo, Japan).

The parameters quantified included the number of blood vessels per field (100x magnification), arteriole wall thickness in proportion to total vessel area, and astrocyte number and activation state.

Angiogenesis was assessed by quantifying the number of blood vessels per field. All identifiable vessels were counted in 15 random non-overlapping 100x magnification fields per section. Vessel counts were performed on Martius Scarlet Blue (MSB) stained slides to enhance vascular delineation and minimise misidentification.

The arteriole wall thickness was measured via Nikon NIS-Ar digital analysis software, calculating the percentage of wall tissue within the whole vessel area; 18 vessels were examined per animal by drawing Regions of Interests (ROIs) differentiating vessel wall vs. vessel lumen with the Nikon-NIS-Ar software.

The groups compared were CKCS’s with SM/VM (*n* = 13) and CKCS’s without SM/VM (*n* = 6).

### 2.4 Statistical analysis

For each metric a mean and standard deviation was calculated. Values for CKCS’ affected by SM/VM/CM were compared to unaffected CKCS’ using non-parametric Mann–Whitney’s U test (GraphPad Prism 11.0.2).

## 3 Results

### 3.1 Blood vessel density and angiogenesis

SM and VM affected CKCS displayed increased blood vessel density compared to the control group. There were large numbers of small blood vessels adjacent to the ventricles, identified by their characteristic circumferential walls, containing a layer of endothelial cells which line a lumen.

**Figure 2:**
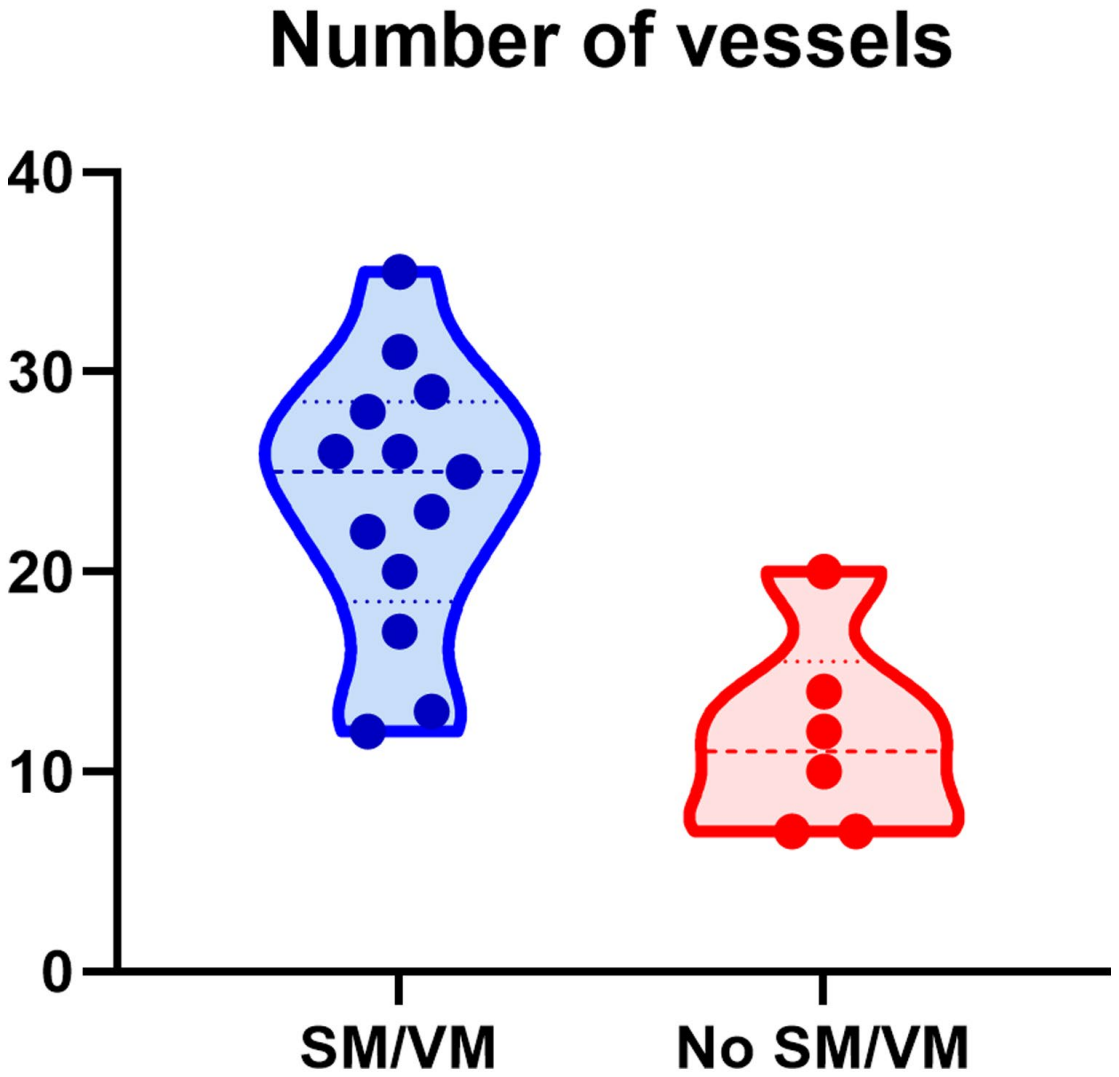
Violin plot showing differences in median blood vessel number calculated over 15 low powered fields (10x magnification). The group affected by SM/VM show an increased number of vessels compared to the control (no SM/VM) suggestive of increased angiogenesis.

In the group affected by SM/VM, samples averaged 23.62 ± 6.79 blood vessels per field, more than twice that of the control group (mean 11.67 ± 4.93 blood vessels per field) (*p* = 0.0019) (Figure 2).

### 3.2 Arteriole wall thickness and special staining

**Figure 3:**
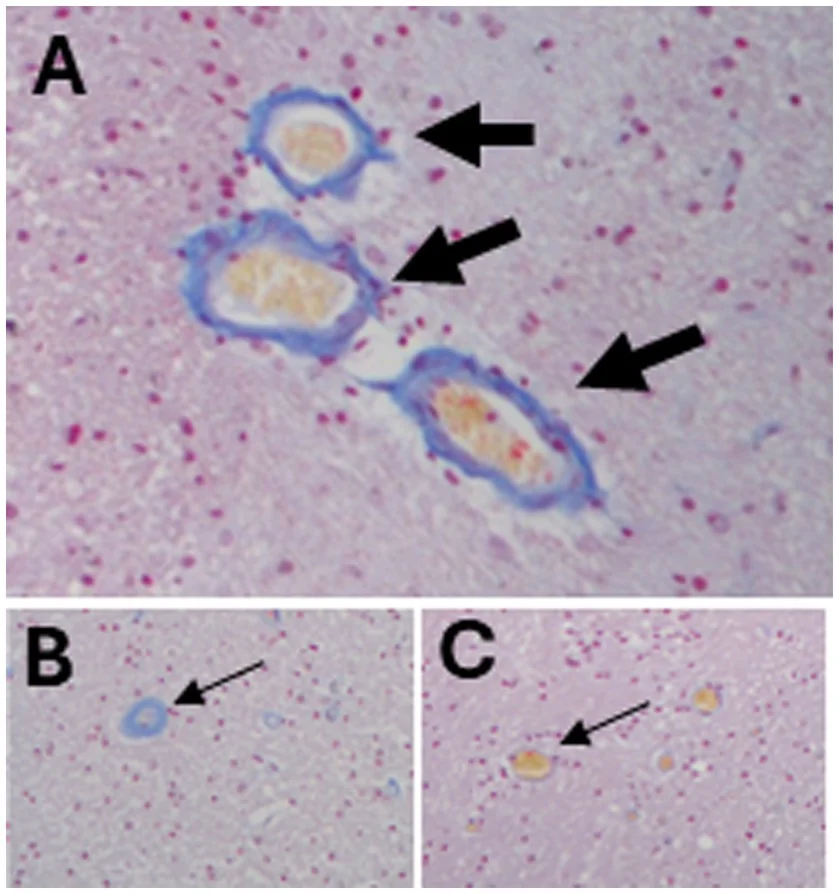
Trichromic Martius Scarlet Blue (MSB) staining of collagen within arteriolar walls (arrows) of the cerebral parenchyma adjacent to lateral ventricle in a canine with SM/VM showing increased thickness **(A,B)** compared to control dog **(C)**. Original magnification: A: 400x, B and C: 100x.

The arteriolar walls in periventricular sections exhibited moderate thickening with luminal narrowing in the CKCS with SM and VM. Special staining with MSB highlighted arterioles and revealed a large extent of collagen deposition within the arteriole walls (Figure 3).

**Figure 4:**
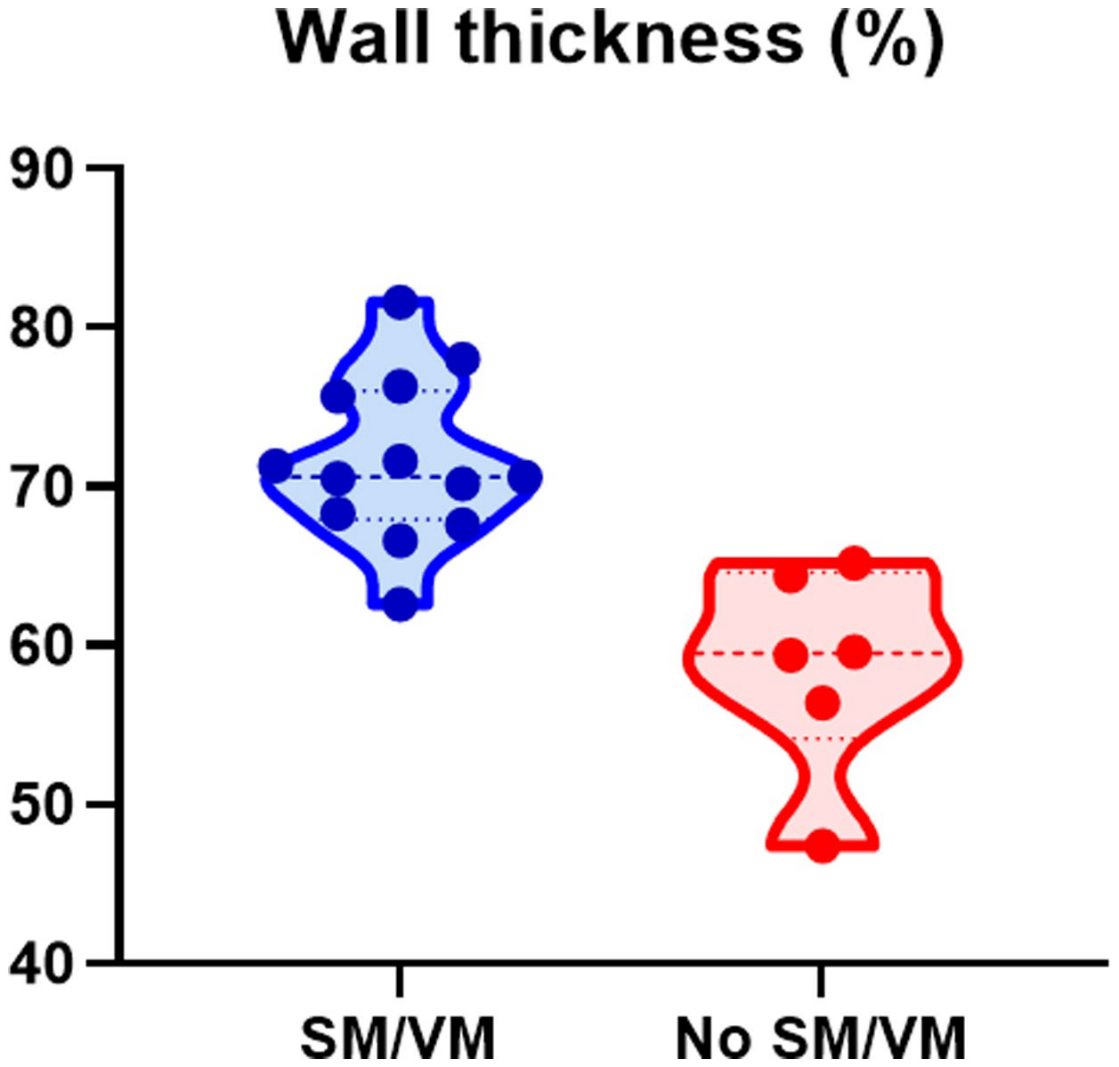
Violin plot presenting results quantifying arteriole wall thickness between the affected group (SM/VM) and the unaffected control group (No SM/VM) calculated as a percentage of total arteriole area within the region of interest by image analysis software. Affected dogs consistently showed thicker arteriole walls than unaffected dogs.

The SM/VM group showed, on average, a 21.9% greater relative arteriolar wall thickness compared with unaffected dogs (71.61% ± 5.14 vs. 58.73% ± 6.47) of total vessel diameter (*p* = 0.0003) (Figure 4).

### 3.3 Astrocytes and GFAP immunohistochemistry

**Figure 5:**
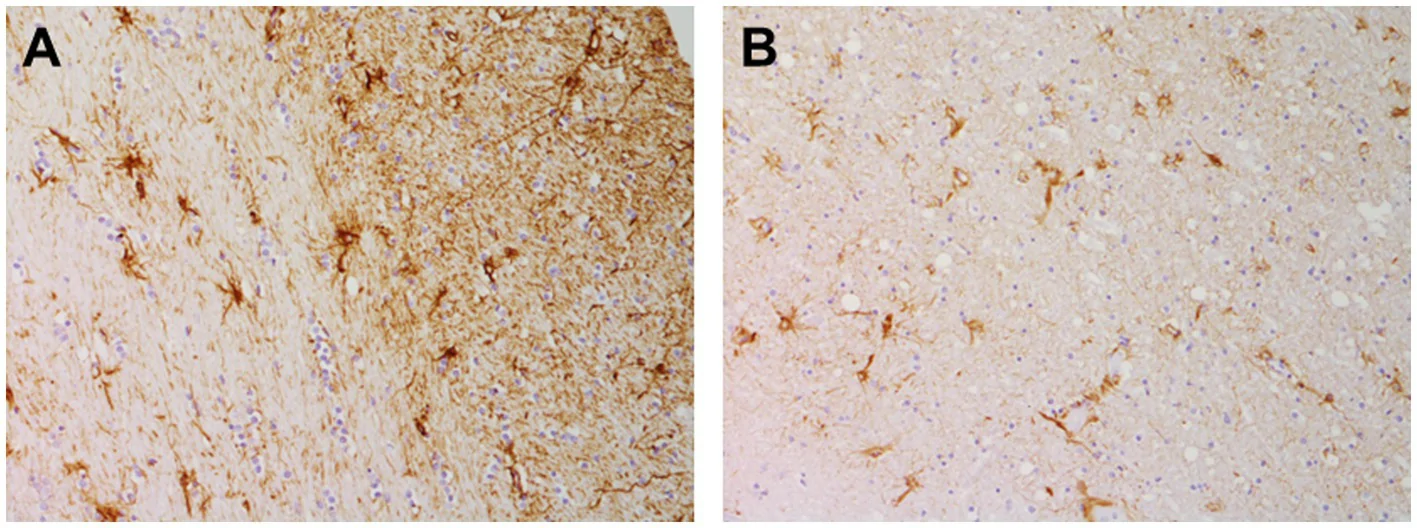
GFAP IHC staining within the cerebral parenchyma adjacent to the lateral ventricles from a canine with SM/VM **(A)** and an unaffected individual **(B)**. GFAP expression is higher in the SM/VM canine, also showing increased cytoplasmatic processes.

In CKCS with SM/VM (Figure 5A), the periventricular tissue displayed comparatively higher numbers of GFAP-positive astrocytes compared with controls (Figure 5B).

Increased GFAP staining was particularly evident immediately adjacent to the ventricles where an increased population of astrocytes was visible. In SM/VM affected individuals, astrocytes had comparably larger cytoplasmic processes and enhanced GFAP immunostaining compared to astrocytes residing in the control population.

## 4 Discussion

The study identified a marked increase in periventricular vascularity, arteriolar wall hypertrophy and astrogliosis via microscopic assessment in CKCS with SM and VM. Despite the presence of VM and these marked periventricular histopathological changes, the dogs included in this study did not exhibit overt clinical signs attributable to forebrain or cerebellovestibular dysfunction. The presence of histopathological alterations despite the lack of clinical signs is an important finding, suggesting that clinically silent ventricular enlargement may still be associated with significant underlying neuropathological changes.

Vascular proliferation within the brain can occur in response to hypoxia, triggered by various angiogenic factors such as VEGF, angiopoietin-2 and erythropoietin (34–38). VEGF and its associated receptor VEGFR2 have been associated with increased blood vessel density, which has been observed in the hippocampal region of the brain in animals with chronic hydrocephalus (25, 39, 40). Although angiogenesis is intended to ameliorate hypoxia to brain tissue, it can have a damaging effect through the blood brain barrier becoming more permeable and the disjointed vascular coupling (41, 42) allowing fluid infiltration into the cerebral parenchyma, possibly worsening existing disease (25). Significant vascular proliferation was observed in the population with SM/VM, more than twice the number of blood vessels seen in the population without SM/VM. The angiogenesis observed is suggestive that the parenchyma surrounding the ventricles is undergoing hypoxic insult and has responded with vascular proliferation presumably secondary to VEGF release.

Our study found that arterioles within the brain had significantly increased wall thickness, visible with the MSB stain. This is suggestive of hypertrophy of the tunica media’s smooth muscle cells and increased production and deposition of collagenous matrix (43–45), possibly in response to increased mechanical stress due to hypertension.

Arterioles make up part of the microvascular bed, delivering oxygenated blood from the terminal arteries to the capillaries; they control resistance to flow in response to physiological stimuli primarily by changing the diameter of their lumens and through this mechanism they regulate tissue perfusion and arterial pressure to an extent (46). A reduction in lumen diameter will markedly increase peripheral resistance in the relaxed vessels (47) and therefore pressure.

Astrocytes are glial cells with a significant role in maintaining brain homeostasis, through the regulation of neuroinflammation and blood brain barrier integrity/permeability, and supporting the function of neurones (48). In response to pathological processes, especially those of a milder and more chronic nature (49, 50), these astrocytes become activated, which can be visualised as enhanced GFAP expression (51, 52). Activation of astroglia initiates production of anti-inflammatory cytokines and facilitation of parenchymal repair (53, 54); however, these cells can produce proinflammatory molecules and neurotoxic substances which may exacerbate the inflammatory response to brain tissue injury (55, 56). These cells also migrate to sites of injury and rapidly proliferate to form a glial scar (57), a scaffold to support areas of parenchymal loss. Although the primary goal of astrocytic activation and its production of inflammatory mediators is to benefit the host, exaggerated or chronic activation can facilitate pathological changes itself to the neuroparenchyma through the exacerbation of neuroinflammation (48). Qualitative analysis of GFAP immunostaining in our study is supportive of astrocytic activation and proliferation, the brain’s response to a variety of possible insults. The activation and proliferation of astrocytes that release cytokines such as VEGF will have a potent effect on vasculature, stimulating angiogenesis (58).

Previous studies have focused on histopathological changes and cytokine signalling present after mechanical injury and hypoxic events, with VEGF signalling playing a key role in the brain’s response to hypoxic/ischaemic events leading to increased blood vessel density (25). The literature also describes fibrous thickening of arteriolar walls in chronic pathological disease states such as hypertension contributing to disturbed blood flow by narrowing lumens and reducing the brain’s capacity to autoregulate, exacerbating cerebral diseases further (59). It is hypothesized that similarities in the pathologic mechanisms involved in normal pressure hydrocephalus and SM exist with key histological changes including periventricular angiogenesis with increased VEGF expression and gliosis (24, 25, 29, 30). Similar pathological changes of angiogenesis, and gliosis in the spinal cord of CKCS affected with SM subsequent to CM is documented accompanied by an increased arteriolar wall thickness adjacent to the syrinx cavity (14) supporting the role of vascular remodelling and compliance in the pathogenesis.

Histopathological investigations of SM have to date primarily focused on tissue immediately surrounding the syrinx cavity (14). In contrast, the present study demonstrates that periventricular brain tissue exhibits changes comparable to those described contralateral to syrinx cavities. These findings suggest that similar pathological mechanisms may be operating more diffusely throughout the central nervous system in SM.

Two principal hypotheses exist regarding syrinx propagation in CM: the arterial pulse mismatch model and the vascular compliance model (60–63). Both models highlight the important interaction between vascular and CSF pulsatility (60–63). However these hypotheses are dissimilar in the key driving mechanism – referring to the mismatched pulse timing between arterial and CSF waves, and impaired vascular compliance with venous congestion, respectively (60–63). The traditional arterial pulse mismatch model emphasises how in health arterial expansion and relaxation within the cerebral and spinal cord parenchyma rhythmically changes intracranial volume; to maintain constant intracranial volume and therefore intracranial pressure, CSF is displaced according to the cardiac cycle either rostrally or caudally. These pressure waves are synchronised in health to minimise increases in intracranial pressure throughout the cardiac cycle (Monro-Kellie doctrine) (64). In Chiari-like malformation, this model theorises that changed cerebral architecture hampers the CSF flow efficiency between cranial and spinal compartments, interfering with pulse pressure synchronicity leading to transient pressure increases within the CSF cranial compartment, forcing CSF down the spinal cord and into the perivascular spaces, forming a syrinx (60, 61). Histologically, according to this model, signs of fluid entering the perivascular space would be expected along with little vascular remodelling due to the cause being hydrodynamic rather than vascular. Although this model is widely cited, histological evidence consistent with perivascular intrusion of CSF and its associated tissue responses are lacking. Alternatively, the vascular compliance model outlines the significance of the vasculature’s role in damping arterial pulsations by the Windkessel Effect to maintain local pressures (62, 63). The reduction of compliance leads to increased localised pressure, vascular leakage and formation of a syrinx. Histologically one would expect to see vascular remodelling and gliosis, a hypoxaemic response secondary to chronic pressure stress.

Our histological and immunohistochemical findings demonstrate arterial wall thickening due to marked collagen deposition, accompanied by gliosis and angiogenesis, consistent with chronic pulsatile stress and hypoxic injury in tissues adjacent to cerebrospinal fluid compartments. This pattern of change is more consistent with the vascular compliance model, as collagen accumulation within arteriolar walls would be expected to impair arterial distensibility, reduce effective damping of pulsatile flow, and result in elevated local pressures with secondary hypoxic injury. This contrasts with the pulse mismatch model, which attributes tissue damage primarily to temporal discordance between arterial pulsation and cerebrospinal fluid dynamics.

Although some features observed in this study, including perivascular stress responses and evidence of tissue hypoxia, are also predicted by the pulse mismatch model, additional findings argue against it as the primary mechanism. Specifically, previous demonstration of cerebrospinal fluid within perivascular spaces of the spinal cord (14), together with clear evidence of impaired arterial compliance and vascular remodelling in both the spinal cord and cerebral parenchyma, supports a mechanism in which vascular pathology drives transvascular fluid filtration into perivascular spaces and contributes to a self-perpetuating cycle of deterioration. The pulse mismatch model does not account for altered vascular compliance as a pathogenic contributor and therefore does not adequately explain the histological changes observed in this study.

Overall, our observations of periventricular angiogenesis, arteriolar wall thickening with collagen deposition, and astroglial changes are compatible with impaired vascular compliance and chronic tissue stress in dogs with SM/VM secondary to CM. While these findings do not provide evidence of causation, they offer a foundation for future studies aimed at characterising the pathological changes associated with disease progression.

## 5 Limitations

This study was limited by a small sample size (*n* = 19) with unequal group sizes (VM/SM = 13; Unaffected by SM/VM = 6) reducing the statistical power and limiting generalisability. Although inherently statistically underpowered, small data sets remain valuable when interpreted alongside existing literature (65), especially the larger studies involving experimentally induced CM. Obtaining appropriate numbers of control dogs (unaffected by SM/VM) is particularly challenging because CM is highly prevalent in CKCSs, with syringomyelia affecting approximately 50% of dogs during their lifetime (66–68) and up to 70% of dogs over 7 years of age (10). Furthermore, obtaining high-quality post-mortem tissue requires rapid retrieval following euthanasia. This depends on owner consent, transport of the dog to an appropriate facility, and immediate post-mortem examination, all of which substantially limit case availability. This study was only possible because of the exceptional commitment of the CKCS community, whose members raised awareness, coordinated logistics, and raised funds to facilitate post-mortem tissue collection. Despite these constraints, naturally occurring post-mortem tissue from well-characterised cases remains a valuable resource for investigating disease mechanisms in both veterinary and comparative neuroscience (65).

Due to the retrospective study design, histopathological assessment was performed only at the end of life, with dogs often representing advanced stages of disease or having concurrent comorbidities, including myxomatous mitral valve disease (MMVD), which could not be excluded. Although MMVD, which CKCS’s are particularly predisposed to (69, 70), represents a potential confounding factor, the neuropathological changes associated with chronic cardiac disease would be expected to reflect the effects of chronic systemic hypoperfusion. Reported cerebral changes associated with MMVD include diffuse neuronal loss, hippocampal injury, white matter degeneration, microglial activation, diffuse gliosis and, in cases of severe heart failure, small vessel disease (70–72). In contrast, the prominent periventricular angiogenesis, collagenous arteriolar remodelling and astrocytosis observed in the present study are not characteristic features of MMVD-associated brain pathology alone. Nevertheless, although these pathological changes were strongly associated with SM/VM, the retrospective nature of this study precludes establishing causality, and the observed alterations may represent consequences of disease progression rather than mechanisms underlying the development of SM/VM. Furthermore, variation in age, disease severity and concurrent comorbidities among the dogs likely contributed to the heterogeneity of the histopathological findings. However, given the rarity of suitable control tissue and the difficulty in obtaining closely matched cases, some degree of heterogeneity was unavoidable.

As this study was restricted to CKCSs, these findings may not be directly applicable to other breeds that CM and SM occurs in, such as the Griffon Bruxellois, or other species, including brachycephalic cats (1). Comparative histopathological studies across affected species and breeds would be informative in determining whether similar pathological mechanisms and histopathological findings are conserved.

Future studies should seek to expand the number of well-characterised cases as opportunities arise, recognising that assembling large, age-matched cohorts is unlikely to be feasible for naturally occurring disease requiring rapid post-mortem tissue collection. Rather than relying solely on larger pathological datasets, greater advances are likely to come from integrating histopathological findings with advanced MRI, fluid biomarkers, and other non-invasive measures that can be repeatedly assessed throughout life. These approaches would allow post-mortem pathology to serve as a reference standard for validating imaging and biomarker signatures, facilitating longitudinal investigation of disease progression without the ethical and practical constraints of repeated tissue sampling (see Future Directions).

## 6 Future directions

The study of CKCSs affected by Chiari-like malformation provides a natural spontaneous model of SM development and its associated features, which cannot easily be matched by experimentally induced disease in various laboratory animals, or sporadic investigations in humans. The relatively large population of cadavers that underwent prompt post-mortem examination and histopathological processing upon death is a significant strength of this study, adding to the knowledge base of CM and SM.

Further evaluation of the cerebral neuroinflammatory response through larger populations with the additional assessment of microg lia and oligodendrocytes peri ventricularly, and within other areas of the brain such as the corpus callosum (73, 74), would provide a more complete picture of the ongoing neuroinflammation underlying CM and its sequelae.

Alongside larger studies with enhanced assessment of various neuroinflammatory changes, future research should aim to correlate the histopathological findings observed in endstage CKCSs with CM to imaging-based features, thereby facilitating translation of cadaveric tissue changes into non-invasive, clinically observable markers. This would likely be done by first imaging the brain and spinal cord of CKCS cadavers prior to sampling and histopathol ogy so findings may be linked. To further support establishing any causal relationships within our histological findings, observation of tissue changes at all stages of life through a non-terminal diag nostic modality, such as advanced imaging, would be of substan tial value. Imaging could be complemented by biomarker assays targeting cytokines and other biomarkers likely involved in the tissue changes we have observed. For example, blood GFAP levels have been shown to be indicative of traumatic brain injury severity (75, 76) and micro-RNA has also been correlated with cerebral injury and may provide a useful monitoring modality (77, 78). Together, these research directions aim to translate histopathologi cal observations into actionable interventions and diagnostics for CM-associated SM.

## Data Availability

The raw data supporting the conclusions of this article will be made available by the authors, without undue reservation.
